# Osteoporosis Prevention and Treatment: The Risk of Comorbid Cardiovascular Events in Postmenopausal Women

**DOI:** 10.7759/cureus.24117

**Published:** 2022-04-13

**Authors:** Zachary A Gilbert, Avia Muller, Jillian A Leibowitz, Marc M Kesselman

**Affiliations:** 1 Osteopathic Medicine, Nova Southeastern University Dr. Kiran C. Patel College of Osteopathic Medicine, Davie, USA; 2 Rheumatology, Nova Southeastern University Dr. Kiran C. Patel College of Osteopathic Medicine, Davie, USA

**Keywords:** osteoporosis treatment, osteoporosis, cardiovascular disease, cardiovascular disease prevention, coronary artery calcium score, calcium supplement, post-menopausal women

## Abstract

Treatment modalities used for the management of postmenopausal osteoporosis have come under increased scrutiny more recently due to the elevated risk of cardiovascular disease (CVD) among this patient population. A review of the literature found that postmenopausal women with osteoporosis were at an increased risk of experiencing cardiovascular events such as myocardial infarction. This increased CVD risk among postmenopausal women with osteoporosis has been linked to the use of calcium supplements. It has also been linked to the presence of sclerostin, a wingless-type mouse mammary virus-integration site pathway, which is currently being used as a target for some osteoporosis medications. Research efforts have demonstrated that the prevention and treatment of osteoporosis, especially among postmenopausal women, need to be carefully designed to prevent and reduce the risk of CVD events. As such, the most effective regimens should be tailored to each patient, ensuring that the benefits of certain treatments, such as selective estrogen receptor modulators and calcium supplementation, outweigh the potential health risks, especially CVD event risk among postmenopausal women.

## Introduction and background

Osteoporosis is a systemic and chronic condition that is characterized by low bone mass density and bone deterioration [1]. The condition is fairly common and impacts a wide variety of individuals, which can result in increased bone fragility and fractures as well as increased morbidity and mortality, especially among postmenopausal women [2,3]. Postmenopausal women experience a decrease in estrogen levels and an increase in bone resorption, affecting their bone density and increasing cardiovascular calcification risk [4]. Experts in the field have outlined strategies for prevention and clinical criteria for diagnosis, as well as highlighted effective treatment options, but guidance on prevention and treatment among postmenopausal women remains limited, especially as it relates to the risk of developing comorbid cardiovascular disease (CVD) [3,5]. It is important to note that while this review focuses on the impact of osteoporosis and its treatment on CVD, CVD shares predisposed and lifestyle risk factors with osteoporosis, such as age, level of activity, and tobacco use [5]. These limitations are not only the result of limited research studies on the developing topic, but also conclusion inconsistencies among different treatment options and their relative cardiovascular risks [6]. Research data suggest that the increased risk of developing CVD complications, especially among postmenopausal women when compared to their male counterparts, is likely associated with the increased progression of abdominal aortic calcification (AAC) tied to bone loss [5]. Arterial calcification correlated to the increased bone resorption seen in osteoporosis can also be assessed using the coronary artery calcification (CAC) score [7]. This score can be used clinically to assess patients’ risks for CVD [7]. This raises the question of whether the benefits of treatment using dietary calcium or calcium supplementation, with and without the use of vitamin D, outweigh the risk of developing cardiovascular complications among postmenopausal women [6]. This review will discuss the current literature regarding CVD risk and postmenopausal women’s osteoporosis prevention and treatment options, such as bisphosphonates, estrogen modulators, parathyroid hormone (PTH) analogs, and antiresorptive calcitonin analogs as an alternative therapy [8].

## Review

Postmenopausal women and risk of CVD events

**Figure 1:**
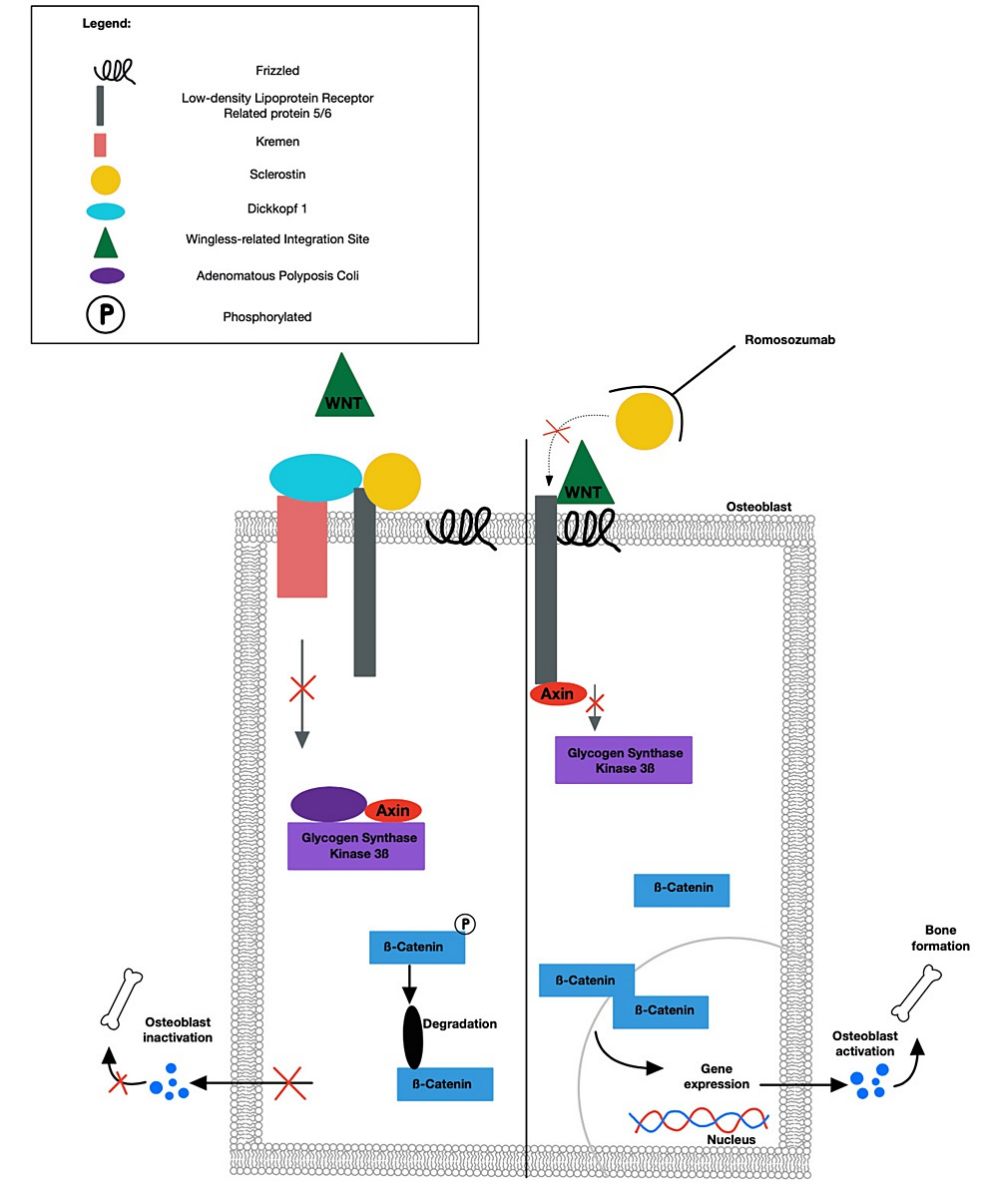
The WNT signaling pathway and the role of sclerostin in the inhibition of the pathway and subsequent bone formation. The right-hand side of the figure demonstrates the role of romosozumab in the inhibition of sclerostin binding, leading to the promotion of the bone formation Figure credit: Zachary Gilbert, Avia Muller, and Jillian Leibowitz.

An increased risk for osteoporosis exists among postmenopausal women, attributable to the presence of decreased estrogen levels, which can lead to increased sensitivity to PTH and subsequent bone resorption [4]. Intrinsically, this can lead to an increased risk of CVD events such as myocardial infarction (MI) [4]. Postmenopausal women diagnosed with osteoporosis have a 47% increased likelihood of developing CVD when compared to those women with normal bone mineral density (BMD) levels [4]. It has been theorized that this elevated risk is associated with the presence of atherosclerosis and calcium interventions used to treat osteoporosis. Sclerostin, which is a naturally occurring inhibitor of the wingless-type mouse mammary virus-integration site (Wnt) pathway, expressed by osteocytes, has also been demonstrated to play a role [9]. The Wnt pathway plays a key role in osteogenesis by regulating the synthesis and survival of osteoblasts [9]. As demonstrated in Figure 1, inhibition of the Wnt pathway by sclerostin prevents bone formation [9,10]. In addition, sclerostin levels are known to have an inverse relationship with BMD readings [6]. Meanwhile, a new study demonstrated a link between sclerostin and its role in the formation of atherosclerotic plaques, predisposing patients who underwent carotid endarterectomy to CVD events [9]. Monoclonal antibodies are currently being evaluated in clinical trials and have demonstrated promising results, increasing BMD in patients with osteoporosis [10]. In a phase III study, romosozumab, a monoclonal IgG2 antibody targeting sclerostin, demonstrated improvements in BMD, while also decreasing markers of bone resorption. Specifically, romosozumab demonstrated a 13.7% increase of BMD in the lumbar spine and a 6.8% increase in the total hip when compared with alendronate and subcutaneous teriparatide [11]. While blosozumab also showed an increase in bone formation, its use has been discontinued due to injection site reactions [11]. The previous study compares the effectiveness in increasing BMD between romosozumab and alendronate, whereas another study compared their associated CVD incidents, concluding that romosozumab was associated with more serious CVD incidents when compared with alendronate, potentially due to targeting sclerostin [9].

Furthermore, postmenopausal women are at an increased risk of osteoporosis due to the amount and distribution of their adipose tissue, which also puts them at a higher risk of CVD events. Studies have shown that, when compared to premenopausal women, postmenopausal women have both an increased total body fat and an increased body fat distribution index [12]. In the same study, a similar correlation was seen between body fat percentage and risk of CVD events such as MI, showing a significant value of <0.001, when compared with premenopausal women [12,13]. This indicates a baseline risk of cardiovascular events in postmenopausal women, even before osteoporotic treatment interventions.

Prevention and treatment of osteoporosis in postmenopausal women and risk of CVD events

Prevention and treatment of osteoporosis, especially among postmenopausal women, need to be carefully designed to prevent and reduce the risk of CVD events [14]. Several prevention and treatment strategies used to reduce the risk for osteoporosis among postmenopausal women include estrogen receptor modulators [15], calcium supplementation [16], dietary calcium [17], vitamin D [7], and cathepsin K inhibitors such as Odanacitib [18]. Cathepsin K is expressed by osteoclasts and serves to degrade bone collagen and reduce bone resorption [18,19]. Odanacitib was studied to determine its effects in postmenopausal women by reducing bone resorption [18]. In one study, evaluating the effectiveness of Odanacitib among postmenopausal women in reducing bone resorption, researchers evaluated the development of CVD events including CVD death, MI, or stroke. The researchers found that Odanacitib reduced the risk of bone fractures, but was associated with increased stroke risk [18]. Further investigation into using Odanacitib as a potential strategy for osteoporosis treatment has been halted.

As women age, their estrogen levels face a downward trend creating a loss of homeostasis between bone resorption and remodeling, decreasing overall BMD [20]. Estrogen, a form of estrogen replacement therapy, may only prove to be beneficial within the first 10 years after menopause when its cardiovascular effects are less significant [21]. Selective estrogen receptor modulators (SERMs) have been used in reducing the risk of breast cancer in postmenopausal osteoporotic women and non-osteoporotic women with a high risk of breast cancer [8]. Specifically, raloxifene, which was Food and Drug Administration approved in 1997 for the prevention of osteoporosis in postmenopausal women, is effective in treating osteoporosis within that patient population. While raloxifene has been shown to decrease the incidence of spinal fractures, it is not effective against non-vertebral fractures [21]. As an agonist, raloxifene increases BMD, while preventing fractures and lowering both serum cholesterol and total low-density lipoprotein levels, while its antagonistic effects occur in the breast [22]. Further analysis of the Multiple Outcomes of Raloxifene Evaluation (MORE) study found that raloxifene reduced CVD risk in postmenopausal women with baseline CVD risk, while not significantly decreasing the risk for coronary events or stroke in the rest of the study population [23]. Meanwhile, in the Raloxifene Use for The Heart (RUTH) trial, raloxifene was associated with increased risks of both venous thromboembolism as well as fatal stroke, with the latter seen in women with an already increased risk at baseline. Overall, the researchers found no statistically significant difference in death from coronary causes, non-fatal MI, or hospitalization for an acute coronary syndrome between raloxifene and placebo [24].

The role of calcium through supplementation or dietary intake in the prevention and treatment of postmenopausal osteoporosis remains debated [25]. Several studies support the notion that an increase in dietary calcium has the potential to provide cardioprotective characteristics for women [7]. The risk for coronary artery disease can be assessed using the CAC score. The score is based on CT scan data that analyzes stable and unstable plaques in the arteries. A score of 400 and above presents a highly likely risk of significant stenosis, while lower scores may indicate mild disease or non-obstructive cardiac disease. The CAC score is a progressing, non-invasive method that has shown a stronger ability to predict clinical events over other risk-factor-based estimates, such as the Framingham estimate [26]. One model, the Multi-Ethnic Study of Atherosclerosis (MESA), demonstrated that over a 10-year follow-up period, there was a positive correlation between a high calcium intake and a low CAC score, specifically if calcium intake was reached through diet. These results were adjusted for both a baseline CAC as well as any calcium that was taken as a supplement. [25]. In addition, a study analyzing the effect of dietary calcium among Korean women showed a sensitive balance between reducing CVD, while simultaneously avoiding adverse risks. The researchers found that 389.8 mg is the optimal amount of dietary calcium that should be ingested to provide maximal protection against CVD [17]. While incorporating higher levels of calcium into the diet is beneficial in preventing heart disease, the same is not necessarily true when using supplements as a source of calcium consumption. The same study concluded that higher oral supplementary calcium levels were tied to increased adverse effects [17]. Furthermore, the MESA study, using models adjusted for dietary calcium versus supplementary calcium, found that those who had an increased level of over-the-counter supplementary calcium inadvertently had a 22% increase in CAC [25]. Similarly, a meta-analysis concluded a 24% increased risk of coronary heart disease in participants taking calcium supplementation compared to the placebo group regardless of adding vitamin D to the supplement. Still, the researchers found that women taking personal, non-protocol calcium supplementation before randomization experienced no increased CVD events with the addition of vitamin D [7].

Meanwhile, the benefits of calcium supplementation may outweigh the risks [27]. Researchers exclusively looked at instances of acute MI among patients taking a calcium supplement as either a monotherapy or in conjunction with vitamin D. They found that women taking oral calcium salt supplements with vitamin D of at least 800 IU experienced both a dose- and duration-dependent decrease in instances of acute MI [27]. The mechanism responsible for this increase in calcium remains unclear but possibly points to the metabolism of the calcium itself [17]. By taking a calcium supplement, the body is essentially receiving a bolus of calcium, which leads to a transient period of hypercalcemia. When compared to that of dietary calcium, this hypercalcemia is associated with a much greater risk of CVD [17]. The difference in hypercalcemia is attributed to the slower digestion rate of dietary calcium due to the presence of fats and other molecules, leading to a slower release of the calcium. On the other hand, supplementation is immediately digested, causing a greater period of hypercalcemia [17,25].

Discussion

Decreased levels of estrogen and greater sensitivity to PTH put postmenopausal women at a higher risk of developing osteoporosis and encountering fracture injuries [4]. The increased rate of bone resorption associated with osteoporosis combined with an increased percentage of adipose tissue in postmenopausal women increases their risk for CVD events [12]. In addition, some prevention and treatment strategies for osteoporosis further increase this CVD event risk [5].

In terms of preventative and treatment strategies, the osteoporotic benefits need to be weighed with the risk of adverse CVD events [9,18,24,25]. While romosozumab showed significant improvement in BMD of the hip and lumbar spine [11], its increased adverse cardiac effects should be taken into consideration when being prescribed [9]. Similarly, although Odanacitib was able to decrease the incidence of fractures, its association with increased stroke resulted in its discontinued use [18]. Estrogen receptor modulators, like raloxifene, appear to be promising for increasing BMD and, therefore, decreasing fractures [21]. Meanwhile, the risk of CVD events tied to raloxifene appears inconsistent [23,24]. Additional research is needed on cardiovascular risk for raloxifene [23,24] and calcium supplementation in combination with vitamin D to minimize inconsistencies and improve treatment options [6,7,27].

Increasing calcium levels, whether through dietary changes or oral supplementation, has also shown to be effective in mitigating the effects of osteoporosis. Dietary calcium consumption has been shown to have greater protective effects against CVD events when compared to calcium supplementation, which is associated with increased CVD event risk [25]. This is likely due to the slower rate of absorption over a longer period in the dietary metabolism of calcium. To prevent the bolus of calcium associated with supplementation, dietary intervention is preferred. Based on the research, increasing dietary calcium should be recommended for postmenopausal osteoporotic women when compared to supplementary calcium [17,25]. In addition, the research reviewed failed to mention the type of calcium supplements studied. It would be useful to know whether calcium carbonate, calcium citrate, or another calcium compound was studied to properly compare the supplemental calcium across all studies. This would also allow for more specific recommendations to patients if dietary calcium needed additional supplementation to reach the goal amount.

Current research suggests the need to refine osteoporosis treatment to reduce the risk of CVD. To provide the best treatment options, it would be beneficial to assess patients’ baseline risk for cardiovascular events [12,23-25], renal disease [4], genetic predispositions, and current calcium supplementation [7]. As seen in Tables 1-2, current treatment guidelines take into account patients’ risk levels and medical history to provide a tailored treatment regimen [8].

**Table 1:** Current pharmacological treatment options for postmenopausal osteoporotic women based on guidelines from the American Association of Clinical Endocrinologists/American College of Endocrinology, Endocrine Society and European Society for Clinical and Economic Aspects of Osteoporosis and Osteoarthritis/International Osteoporosis Foundation [8] PTH, parathyroid hormone; CVD, cardiovascular disease; SERM, selective estrogen receptor modulator. Table credit: Avia Muller.

Drug	Mechanism	Clinical use
Alendronate	Bisphosphonate-inhibiting osteoclast activity	Vertebral and non-vertebral fractures, not recommended for patients with CrCl <35 mL/min, initial choice for high-risk groups
Raloxifene	SERM	Prevention and treatment of vertebral fractures, additionally, shown to decrease the risk of breast cancer
Calcitonin	Antiresorptive agent	Alternative agent for acute vertebral fractures, best given in combination with stronger antiresorptive agents
Estrogen	Antiresorptive agent	Administer at the lowest dose and shortest duration necessary, reserved for significant risk groups where alternate treatments are not appropriate
Denosumab	Monoclonal antibody against receptor activator of nuclear factor-kB ligand (RANkL)-inhibiting osteoclast activity	Transition with bisphosphonate after denosumab due to increased risk of vertebral fractures associated with drug discontinuation, initial choice for high- and very high-risk groups
Abaloparatide	Modified PTH-related peptide	Vertebral and non-vertebral fractures, limited for up to two years, initial choice for very high-risk groups
Teriparatide	Synthetic PTH	Vertebral and non-vertebral fractures, limited for up to two years, risk of osteosarcoma, initial choice for very high-risk groups
Romosozumab	Monoclonal antibody against sclerostin increasing osteoblast activity	Vertebral and non-vertebral fractures, should not be considered in women with CVD, initial choice for very high-risk groups

**Table 2:** Current non-pharmacological treatment options for postmenopausal osteoporotic women based on guidelines from the American Association of Clinical Endocrinologists/American College of Endocrinology, Endocrine Society and European Society for Clinical and Economic Aspects of Osteoporosis and Osteoarthritis/International Osteoporosis Foundation [8] Table credit: Avia Muller.

Screenings
1200 mg/day of calcium for women ≥50 years old
1000-2000 IU/day of vitamin D3
Alcohol intake <2 servings/day
Limited caffeine intake
Tobacco smoking cessation
Weight-bearing exercise for 30 min/day

## Conclusions

The increased risk of CVD should be taken into consideration when providing a treatment regimen for osteoporotic postmenopausal women. Sclerostin-targeted drugs, SERMs, and oral calcium supplements have been associated with certain CVD risks such as MI and ischemic stroke. However, other treatment options, such as those that include the use of vitamin D have not shown significant CVD risk and in some instances have even shown a decrease. Current literature recommends assessing each patient’s cardiac history, risk factors, and fracture type, whether vertebral or non-vertebral, to individualize the treatment. Some studies show inconsistencies in their conclusions on the CVD risk of current treatments, specifically raloxifene and combined supplementation of vitamin D and calcium. It is also important to note the source of calcium supplementation, as dietary supplementation was shown to be more protective against CVD. More in-depth research needs to be conducted to study the impact of current treatment regimens on a patient’s CVD risk to achieve more conclusive, confident decision-making between the physician and the patient.
